# Antiplatelet therapy and central nervous system hematomas: a cohort study using real-world data from the FAERS and VigiAccess databases

**DOI:** 10.1097/JS9.0000000000003421

**Published:** 2025-09-08

**Authors:** Lei Wang, Haixia Cai, Shujuan Zhao

**Affiliations:** Department of Pharmacy, Henan Provincial People’s Hospital, People’s Hospital of Zhengzhou University, School of Clinical Medicine, Henan University, Zhengzhou, Henan, China

**Keywords:** antiplatelet drugs, central nervous system, FAERS, hematomas, pharmacovigilance, VigiAccess

## Abstract

**Background:**

Antiplatelet therapy is a cornerstone in the management of atherosclerotic cardiovascular disease. However, the risk profile of central nervous system (CNS) hematomas associated with antiplatelet agents remains incompletely characterized.

**Methods:**

We analyzed CNS-related hematoma adverse event (hAE) reports across the four antiplatelet drugs, using data from the US Food and Drug Administration Adverse Event Reporting System (FAERS) and the World Health Organization’s VigiAccess databases. Disproportionality analysis was conducted to identify positive signals. Stratified analysis assessed risk differentials by age and gender, time-to-onset analysis characterized temporal patterns, and a global assessment of the evidence was established.

**Results:**

A total of 2274 CNS-related hAE reports were identified in FAERS and 7229 in VigiAccess. All four antiplatelet drugs demonstrated significant disproportionality signals, with clopidogrel [FAERS: reporting odds ratio (ROR), 26.79; VigiAccess: ROR: 36.69] and aspirin (FAERS: ROR, 22.06; VigiAccess: ROR, 40.13) showing the strongest associations, followed by prasugrel (FAERS: ROR, 16.91; VigiAccess: ROR, 24.02), and ticagrelor (FAERS: ROR, 8.07; VigiAccess: ROR, 9.80). Subdural hematomas were the most frequently reported subtype (FAERS: 63.11%; VigiAccess: 62.72%). Female patients exhibited stronger signals than males across all drugs. All antiplatelet drugs revealed early failure-type temporal profiles, with median onset times ranging from 12.0 days for ticagrelor to 442.5 days for aspirin (*P* < 0.001).

**Conclusions:**

We found a disproportionately significant association between antiplatelet therapy and CNS-related hematomas, with distinct patterns observed across drug types, patient demographics, and temporal profiles. These findings provide critical insights to inform risk stratification, clinical decision-making, and safety monitoring in patients undergoing antiplatelet therapy.

## Introduction

Antiplatelet agents are cornerstones in the prevention and treatment of atherosclerotic cardiovascular disease, with established efficacy in reducing thrombotic events across various clinical scenarios^[1,2]^. Aspirin, clopidogrel, ticagrelor, and prasugrel are among the most widely prescribed antiplatelet agents, each possessing distinct pharmacological properties and clinical indications^[3]^. However, the inherent mechanism of inhibiting platelet aggregation to these agents increases the risk of bleeding, among which central nervous system-related hemorrhages represent one of the most devastating complications, frequently resulting in substantial morbidity and mortality^[4,5]^.

**HIGHLIGHTS**

- Antiplatelet drugs have been implicated in increasing the risk of central nervous system (CNS)-related hematomas.
- Subdural hematoma was the predominant type of CNS-related hematomas following antiplatelet therapy.
- Stratified analysis revealed stronger signals in females and distinct age-related patterns.
- CNS-related hematomas tended to occur early in treatment and diminished over time.
- Clinicians should maintain vigilance for CNS-related hematomas risk, particularly during the initial treatment period, and consider demographic factors.

Despite its clinical relevance, real-world data on central nervous system-related hematomas following antiplatelet therapy remain limited. Current evidence primarily stems from randomized controlled trials (RCTs), which often exclude patients at high risk for bleeding and, as a result, may fail to capture rare but severe adverse events^[6]^. Furthermore, RCTs frequently report composite bleeding endpoints without detailed characterization of bleeding subtype, thereby limiting their applicability to individual risk assessment^[7]^. Prior meta-analyses have suggested an elevated risk of intracranial hemorrhage with antiplatelet therapy, with relative risk estimates ranging from 1.13 to 2.50 compared to placebo or control therapy^[8,9]^. However, these analyses have predominantly focused on aspirin or older agents, providing less comprehensive data on newer agents such as ticagrelor and prasugrel. Additionally, few studies differentiate intracranial hemorrhage subtypes (e.g., subdural, epidural, and intraparenchymal) or explore potential risk variations by demographic characteristics or time to onset^[10]^.

To address this knowledge gap, the present study aims to provide a comprehensive analysis of central nervous system-related hematomas following antiplatelet therapy using real-world, large-scale data from global pharmacovigilance databases. We aim to: (1) assess the strength of the association between central nervous system-related hematomas and the four widely used antiplatelet agents (aspirin, clopidogrel, ticagrelor, and prasugrel); (2) characterize the subtypes of central nervous system-related hematomas reported for each agent; (3) identify potential demographic risk modifiers; (4) analyze the temporal patterns of central nervous system-related hematoma events; and (5) establish global assessment between our findings and existing evidence.

## Methods

### Data sources and processing procedure

We extracted central nervous system (CNS)-related hematoma adverse event (hAE) reports from two major pharmacovigilance databases: the US Food and Drug Administration (FDA) Adverse Event Reporting System (FAERS) database, and the World Health Organization’s global database VigiAccess. The FAERS database covers reports from 2004 Q1 to 2024 Q4, while VigiAccess includes reports from the drug’s market introduction to 29 December 2024. FAERS is a comprehensive database containing spontaneous adverse event (AE) reports submitted by health care professionals, consumers, and drug manufacturers globally^[11]^. VigiAccess, managed by the Uppsala Monitoring Centre, allows access to drug safety reports received worldwide^[12]^.

In this study, CNS-related hAEs were defined as any hematoma events occurring within the central nervous system, including the brain and spinal cord, reported in association with the use of antiplatelet agents. The cases were classified using the preferred terms (PTs) from the Medical Dictionary for Regulatory Activities (MedDRA) version 27.1 (Supplementary Digital Content, Table S1, available at: http://links.lww.com/JS9/F20)^[13]^. The four antiplatelet agents included in this study were aspirin, clopidogrel, ticagrelor, and prasugrel, chosen for their widespread clinical use and distinct mechanisms of platelet inhibition. To ensure a potential association between drug exposure and the reported hematoma event, only cases where the antiplatelet drug was reported as the primary suspect in the AE report were included. This work has been reported in line with the STROCSS criteria^[14]^. No artificial intelligence tools were used in any aspect of the study design, data analysis, manuscript preparation, or editing process.

Additionally, warfarin was used as a positive control, as its association with CNS-related hemorrhage is well-established. Rosuvastatin was selected as a negative control, given its frequent co-prescription with antiplatelet agents in cardiovascular patients, yet lacking documented hemorrhagic risk in product labeling or clinical studies.

Based on the established guidelines and the removal criteria^[15]^, we performed data deduplication to eliminate duplicate reports. After deduplication and exclusion steps, a total of 2274 CNS-related hAE reports associated with the four antiplatelet drugs were identified in the FAERS database, while 7229 reports were identified in the VigiAccess database.

### Descriptive analysis

We performed a descriptive analysis of patient characteristics from CNS-related hAE reports following antiplatelet therapy. Key variables examined included gender, age, age group, reporter type, reported countries, seriousness of events, outcomes, and reporting year. Serious outcomes were classified according to regulatory definitions, including life-threatening events, hospitalization, disability, death, congenital anomalies, required intervention, and other significant events. For this study, the descriptive analysis was limited to the FAERS database. While VigiAccess provides valuable information on AE frequencies and signal detection metrics, the publicly accessible version lacks the detailed demographic data necessary for a comprehensive analysis of specific AEs.

### Disproportionality analysis

Signal detection was performed using four disproportionality algorithms: reporting odds ratio (ROR), proportional reporting ratio (PRR), Bayesian confidence propagation neural network (BCPNN), and multi-item gamma Poisson shrinker (MGPS)^[16–19]^. These methods identify potential positive signals by comparing the target events and target drugs to all other events and drugs, using a four-grid table calculation method (Supplementary Digital Content, Table S2, available at: http://links.lww.com/JS9/F20).

The parameters for AE signal detection were defined as follows: (1) for ROR, when *a* ≥ 3 and the lower bound of the 95% confidence interval (CI) > 1; (2) for PRR, when *a* ≥ 3 and the lower bound of the 95% CI > 1; (3) for BCPNN, when the lower limit of the information component (IC025) > 0, with values between 0 and 1.5 categorized as weak signals, between 1.5 and 3 as medium signals, and >3 as strong signals; and (4) for MGPS, when the lower limit (EBGM05) of the 95% CI for the empirical Bayes geometric mean (EBGM) > 2, with *a* > 0 (Supplementary Digital Content, Table S3, available at: http://links.lww.com/JS9/F20). In these disproportionality methods, “*a*” represents the number of occurrences of the target adverse event for the target drug. In our study, antiplatelet drugs were considered to have a significant association with CNS-related hematomas only when the criteria for all four disproportionality methods were met simultaneously.

The disproportionality analysis was conducted in the following steps: (1) a broad evaluation of CNS-related hematomas across antiplatelet drugs was performed using all four algorithms; (2) the frequency and intensity of CNS-related hematoma signals were analyzed at the PT level; (3) to explore potential demographic risk factors, stratified analyses were conducted by age and gender.

### Time-to-onset (TTO) analysis

To analyze the temporal patterns of CNS-related hematomas following antiplatelet therapy, we performed TTO analysis using data from the FAERS database. In this analysis, TTO was defined as the interval between EVENT_DT (the date of occurrence of the AE) and START_DT (the date when the drug was initiated). We employed the Weibull shape parameter test to assess the time to onset of CNS-related hematomas with antiplatelet drugs, focusing on the median, quartiles, and distribution parameters α (scale) and β (shape).

The Weibull shape parameter test includes two components: the scale parameter (α), which defines the scale of the distribution function; and the shape parameter (β), which characterizes its shape. (1) if β < 1 and the upper limit of the 95% CI is < 1, the frequency of hematomas increases initially but decreases over time, indicating an early failure type; (2) if β > 1 and the lower limit of the 95% CI is > 1, the frequency of hematomas increases progressively over time, suggesting a wear-out failure type; (3) if β ≈ 1 and the 95% CI includes 1, it indicates a constant frequency of hematomas throughout the treatment period, classified as a random failure type^[20]^. Cumulative distribution curves were used to visualize the TTO patterns of CNS-related hematomas for different antiplatelet therapies.

### Global assessment of the evidence

The likelihood of potential causality associations between antiplatelet drugs and CNS-related hematomas was assessed using the adapted Bradford Hill criteria, which are widely utilized in epidemiological and pharmacovigilance research^[21–23]^. Our analysis included several key aspects to evaluate the strength and plausibility of the observed associations.

The biological plausibility assessment examined whether a pharmacological mechanism supported the relationship between antiplatelet drugs and CNS-related hematomas. The strength of evidence was assessed by analyzing the magnitude of disproportionality signals, while consistency was evaluated by comparing findings across databases and conducting subgroup analyses. Specificity was used to determine whether certain types of CNS-related hematomas were more strongly associated with specific antiplatelet agents. Temporal patterns were conducted to evaluate whether the sequence and timing of events were consistent with expected pharmacological effects. Additionally, comparisons with other drug classes served as controls to further support our conclusions. Due to the inherent limitations of spontaneous reporting systems and the observational nature of the research design, the rechallenge and dechallenge criteria were not fully evaluated in this study.

### Statistical analyses

Categorical variables were presented as frequencies and percentages, while continuous variables with non-normal distributions were reported as medians with interquartile ranges (IQRs). In the TTO analysis, the Kruskal–Wallis test was used to compare median times of CNS-related hematoma onset across different antiplatelet drugs, with a *P* value <0.05 considered statistically significant.

We conducted a post-hoc power analysis. With the sample size of 2274 CNS-related hematoma adverse event reports in the FAERS database and 7229 reports in the VigiAccess database, we calculated the study’s power to detect various effect sizes at a significance level of 0.05. For disproportionality analyses, we achieved a power of >95% to detect signals with the lower limit of the 95% CI of ROR >1, which is considered clinically significant in pharmacovigilance research. Even for analysis with smaller sample sizes, our study maintained adequate power (>90%) to detect meaningful safety signals. This confirms that our sample size was sufficient to identify clinically relevant associations between antiplatelet drugs and CNS-related hematomas.

Data processing, statistical analyses, and visualizations were conducted using SAS software version 9.4 (SAS Institute Inc., Cary, NC), R software version 4.4.2 (R Foundation for Statistical Computing, Vienna, Austria), and Prism version 9.5 (GraphPad Software, San Diego, CA, USA).

### Ethical statement

This study utilized publicly available data from the FAERS and VigiAccess databases, both of which contain fully anonymized and de-identified patient information. Formal ethical approval and informed consent were not required for this research. This exemption was granted based on the following considerations: (1) all data were obtained from publicly accessible repositories containing no personally identifiable information; (2) these databases were created under appropriate regulatory oversight to ensure patient privacy; and (3) our analysis posed no risk to individual subjects as we had no access to identifying information.

## Results

### Descriptive analysis

From 2004 Q1 to 2024 Q4, a total of 2221 patients reported CNS-related hAEs associated with four antiplatelet drugs in the FAERS database. Clopidogrel (*n* = 1049, 47.23%) and aspirin (*n* = 931, 41.92%) accounted for the majority of cases. Males were predominantly affected (57.81%) across all antiplatelet agents, with the highest proportions observed in the ticagrelor (69.01%) and prasugrel (66.67%) groups. The median age of patients was 75 years (IQR: 66–81), with the majority (68.48%) aged ≥65 years. Notably, the clopidogrel group had the highest proportion of elderly patients (70.92%). Health care professionals reported the majority of cases (78.61%), with physicians being the primary reporters (36.15%), particularly for ticagrelor (77.46%). Geographically, the United States contributed the highest proportion of reports (34.04%), followed by France (14.09%) and Italy (9.99%). Almost all cases were classified as serious (99.82%), with hospitalization (66.64%) and death (23.41%) being the most common outcomes. The report proportions showed an increasing trend over time, with 44.57% of cases occurring between 2019 and 2024, particularly for clopidogrel (61.87%) (Table 1).

**Table 1:** Patient characteristics of CNS-related hAE reports across different antiplatelet drugs in the FAERS database

Characteristics	Total (%)	Aspirin (%)	Clopidogrel (%)	Ticagrelor (%)	Prasugrel (%)
Number of patients	2221 (100)	931 (41.92)	1049 (47.23)	142 (6.39)	99 (4.46)
Gender					
Female	716 (32.23)	313 (33.62)	332 (31.65)	41 (28.87)	30 (30.30)
Male	1,284 (57.81)	551 (59.18)	569 (54.24)	98 (69.01)	66 (66.67)
Not specified	221 (9.95)	67 (7.20)	148 (14.11)	3 (2.11)	3 (3.03)
Age (years)					
Median (IQR)	75 (66–81)	75 (65–81)	76 (69–83)	69 (61–78)	65 (56–73)
<18 years	12 (0.54)	12 (1.29)	0 (0.00)	0 (0.00)	0 (0.00)
18–44 years	55 (2.48)	36 (3.87)	9 (0.86)	2 (1.41)	8 (8.08)
45–64years	335 (15.08)	144 (15.47)	118 (11.25)	40 (28.17)	33 (33.33)
≥65 years	1,521 (68.48)	649 (69.71)	744 (70.92)	84 (59.15)	44 (44.44)
Not specified	298 (13.42)	90 (9.67)	178 (16.97)	16 (11.27)	14 (14.14)
Reporter					
Physician	803 (36.15)	230 (24.70)	437 (41.66)	110 (77.46)	26 (26.26)
Pharmacist	486 (21.88)	135 (14.50)	324 (30.89)	10 (7.04)	17 (17.17)
Other health-professional	457 (20.58)	314 (33.73)	136 (12.96)	3 (2.11)	4 (4.04)
Consumer	398 (17.92)	202 (21.70)	131 (12.49)	13 (9.15)	52 (52.53)
Not specified	66 (2.97)	43 (4.62)	17 (1.62)	6 (4.23)	0 (0.00)
Lawyer	11 (0.50)	7 (0.75)	4 (0.38)	0 (0.00)	0 (0.00)
Reported countries (Top 5)					
United States	756 (34.04)	439 (47.15)	235 (22.40)	33 (23.24)	49 (49.49)
France	313 (14.09)	15 (1.61)	231 (22.02)	50 (35.21)	17 (17.17)
Italy	222 (9.99)	106 (11.39)	111 (10.58)	2 (1.41)	3 (3.03)
United Kingdom	194 (8.73)	51 (5.48)	138 (13.16)	3 (2.11)	2 (2.02)
Japan	139 (6.26)	77 (8.27)	44 (4.19)	0 (0.00)	18 (18.18)
Degree					
Serious	2217 (99.82)	930 (99.89)	1048 (99.90)	141 (99.30)	98 (98.99)
Non-serious	4 (0.18)	1 (0.11)	1 (0.10)	1 (0.70)	1 (1.01)
Outcomes^a^					
Life-threatening	358 (16.12)	106 (11.39)	183 (17.45)	44 (30.99)	25 (25.25)
Hospitalization	1,480 (66.64)	632 (67.88)	708 (67.49)	76 (53.52)	64 (64.65)
Disability	134 (6.03)	34 (3.65)	73 (6.96)	21 (14.79)	6 (6.06)
Death	520 (23.41)	211 (22.66)	239 (22.78)	46 (32.39)	24 (24.24)
Congenital anomaly	0 (0.00)	0 (0.00)	0 (0.00)	0 (0.00)	0 (0.00)
Required intervention	21 (0.95)	7 (0.75)	10 (0.95)	2 (1.41)	2 (2.02)
Other	1,020 (45.92)	359 (38.56)	532 (50.71)	67 (47.18)	62 (62.63)
Reporting year					
2004–2008	131 (5.90)	69 (7.41)	62 (5.91)	0 (0.00)	0 (0.00)
2009–2013	258 (11.62)	127 (13.64)	74 (7.05)	21 (14.79)	36 (36.36)
2014–2018	842 (37.91)	451 (48.44)	264 (25.17)	84 (59.15)	43 (43.43)
2019–2024	990 (44.57)	284 (30.51)	649 (61.87)	37 (26.06)	20 (20.20)

^a^ Some report cases generated >1 adverse event outcome.

CNS, central nervous system; FAERS, the US Food and Drug Administration Adverse Event Reporting System; hAE; hematoma adverse event; IQR, interquartile range.

### Disproportionality analysis

**Figure 1.**
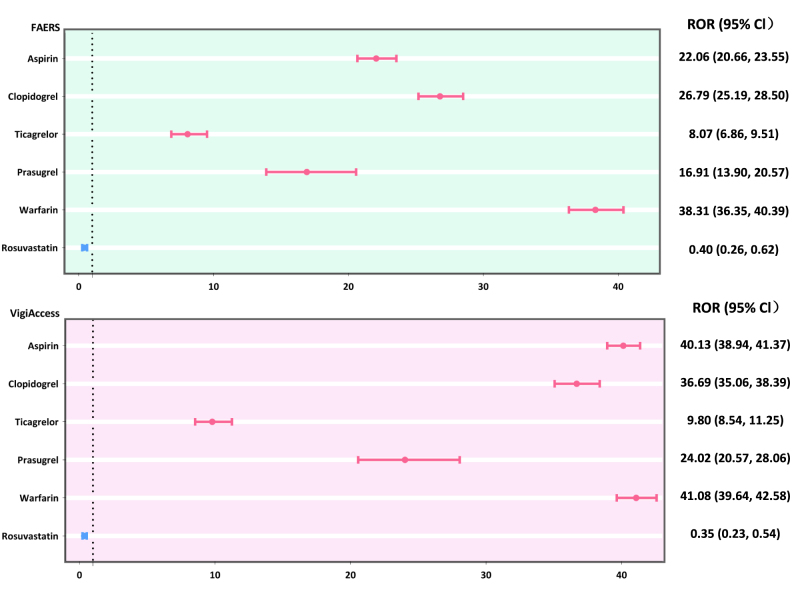
Forest plot of RORs with 95% CIs of CNS-related hematomas across different antiplatelet drugs in the primary analysis.

CI, confidence interval; CNS, central nervous system; FAERS, the US Food and Drug Administration Adverse Event Reporting System.

Disproportionality analysis identified significant safety signals for CNS-related hematomas across all four antiplatelet agents in both databases (Supplementary Digital Content, Table S4, available at: http://links.lww.com/JS9/F20; Figure 1). In the FAERS database, clopidogrel exhibited the strongest signal (ROR 26.79, 95% CI 25.19–28.50), followed by aspirin (ROR 22.06, 95% CI 20.66–23.55), prasugrel (ROR 16.91, 95% CI 13.90–20.57), and ticagrelor (ROR 8.07, 95% CI 6.86–9.51). These findings were consistently supported by all four detection algorithms (ROR, PRR, BCPNN, and MGPS).

Similar patterns were observed in the VigiAccess database, although signal strengths were generally higher. Aspirin showed the strongest signal (ROR 40.13, 95% CI 38.94–41.37), followed by clopidogrel (ROR 36.69, 95% CI 35.06–38.39) and prasugrel (ROR 24.02, 95% CI 20.57–28.06). Ticagrelor maintained the lowest signal (ROR 9.80, 95% CI 8.54–11.25). The BCPNN analyses further validated these findings, with IC025 values >3 for almost all antiplatelet drugs in both databases, indicating robust safety signals.

As expected, warfarin (positive control) demonstrated strong positive signals in both the FAERS (ROR 38.31, 95% CI 36.35–40.39) and VigiAccess (ROR 41.08, 95% CI 39.64–42.58) databases. In contrast, rosuvastatin (negative control) showed no significant signals in either database (FAERS: ROR 0.40, 95% CI 0.26–0.62; VigiAccess: ROR 0.35, 95% CI 0.23–0.54).

### PT-level analysis

**Figure 2.**
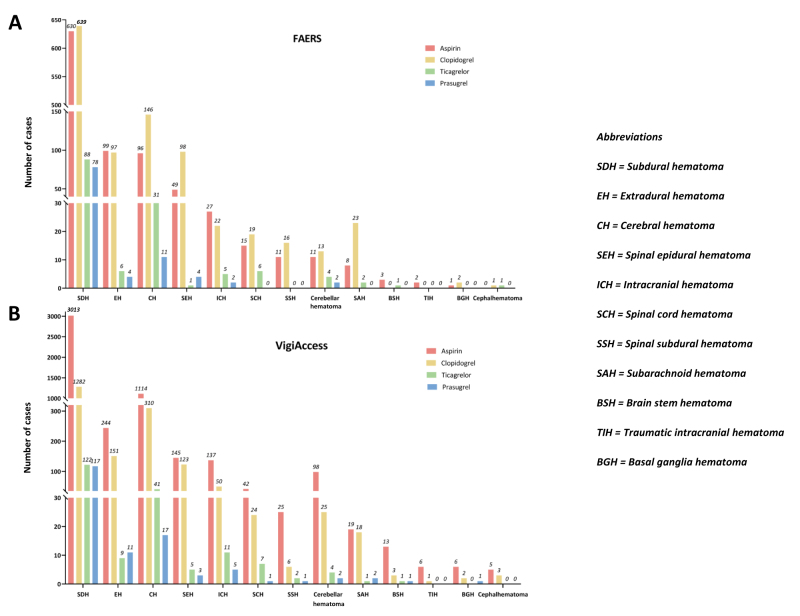
Number of cases of PTs for CNS-related hematoma reports across the four antiplatelet drugs in the two databases. (A) FAERS database. (B) VigiAccess database.

CNS, central nervous system; FAERS, the US Food and Drug Administration Adverse Event Reporting System; PT, preferred term.

At the PT level, subdural hematoma was the most frequently reported CNS-related hematoma across all four antiplatelet drugs in both databases (FAERS: *n* = 1435, 63.11%; VigiAccess: *n* = 4534, 62.72%) (Supplementary Digital Content, Table S5, available at: http://links.lww.com/JS9/F20; Figure 2). In the FAERS database, cerebral hematoma (*n* = 284, 12.49%) and extradural hematoma (*n* = 206, 9.06%) were the second and third most frequently reported PTs, respectively. Similarly, in the VigiAccess database, cerebral hematoma (*n* = 1482, 20.50%) and extradural hematoma (n = 415, 5.74%) maintained the second and third rankings.

**Figure 3.**
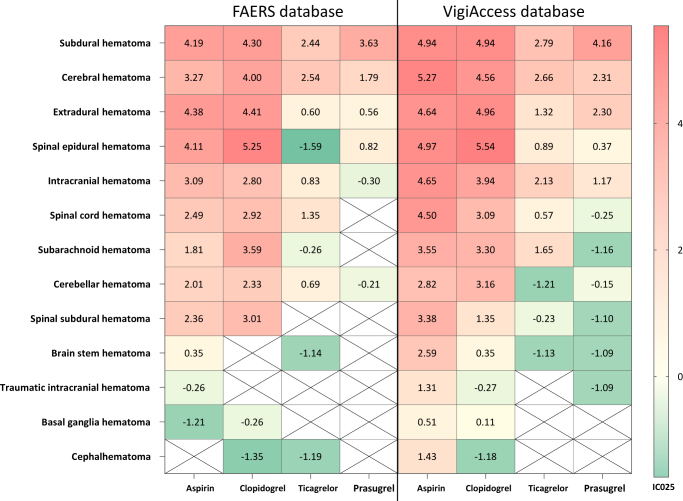
Heat map of the disproportionality analysis at the PT level for CNS-related hematomas across the four antiplatelet drugs in the two databases.

CNS, central nervous system; IC, information component; PT, preferred term.

When applying the BCPNN criteria for signal detection at the PT level, aspirin and clopidogrel exhibited significant positive signals for most CNS-related hematoma subtypes in both databases (Supplementary Digital Content, Table S6, available at: http://links.lww.com/JS9/F20; Figure 3). Specifically, in FAERS, aspirin demonstrated the strongest signal for extradural hematoma (IC025, 4.38), while in VigiAccess, aspirin showed the highest signal strength for cerebral hematoma (IC025, 5.27). Clopidogrel demonstrated the strongest signals for spinal epidural hematoma in both databases (FAERS: IC025, 5.25; VigiAccess: IC025, 5.54).

In FAERS, ticagrelor exhibited the strongest signal for cerebral hematoma (IC025, 2.54), while prasugrel showed the highest signal for subdural hematoma (IC025, 3.63). In VigiAccess, both ticagrelor (IC025, 2.79) and prasugrel (IC025, 4.16) displayed the strongest signals for subdural hematoma.

### Stratified analysis

Gender-specific analysis revealed stronger signal strengths in females compared to males across all four antiplatelet drugs. Clopidogrel showed the most pronounced gender difference, with females exhibiting a significantly higher signal (ROR 31.68, 95% CI 28.38–35.37) compared to males (ROR 19.72, 95% CI 18.14–21.44).

In terms of age groups, aspirin was the only drug that demonstrated positive signals in the pediatric population (ROR 22.57, 95% CI 12.68–40.19). The 18–44 years cohort exhibited particularly strong signals for prasugrel (ROR 133.20, 95% CI 66.05–268.65), while in the 45–64 years group, aspirin (ROR 26.17, 95% CI 22.17–30.89) and prasugrel (ROR 29.70, 95% CI 21.26–41.48) showed the strongest associations. In the elderly population (≥65 years), signals were attenuated but remained significant. Clopidogrel (ROR 13.86, 95% CI 12.88–14.93) and aspirin (ROR 13.48, 95% CI 12.46–14.58) maintained the strongest associations, followed by prasugrel (ROR 8.15, 95% CI 6.06–10.98), while ticagrelor exhibited the weakest signal (ROR 4.57, 95% CI 3.70–5.66).

**Figure 4.**
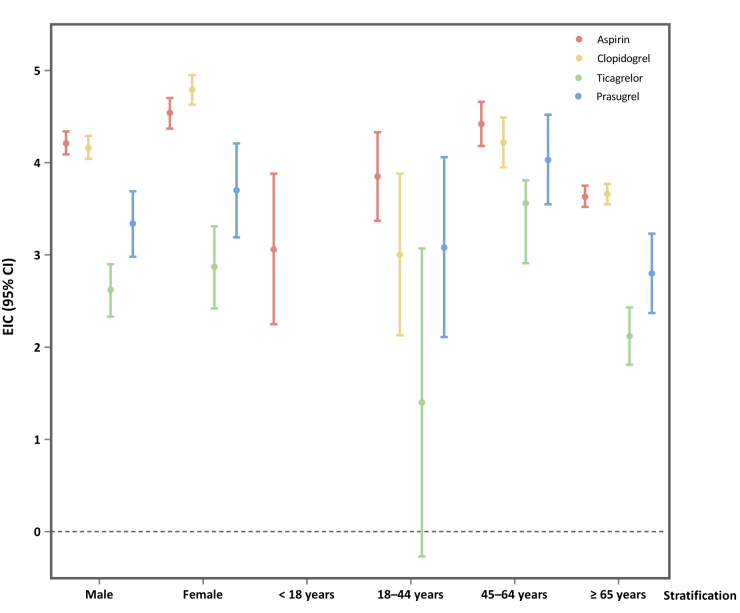
Forest plot displays EIC values of CNS-related hematomas by age and gender across different antiplatelet drugs in the FAERS database, with error bars representing the 95% CI of the EIC.

CI, confidence interval; CNS, central nervous system; EIC, expected information component; FAERS, the US Food and Drug Administration Adverse Event Reporting System.

Almost all detected signals were consistently supported across the four disproportionality detection algorithms (ROR, PRR, BCPNN, and MGPS) (Supplementary Digital Content, Table S7, available at: http://links.lww.com/JS9/F20; Figure 4).

### TTO analysis

**Figure 5.**
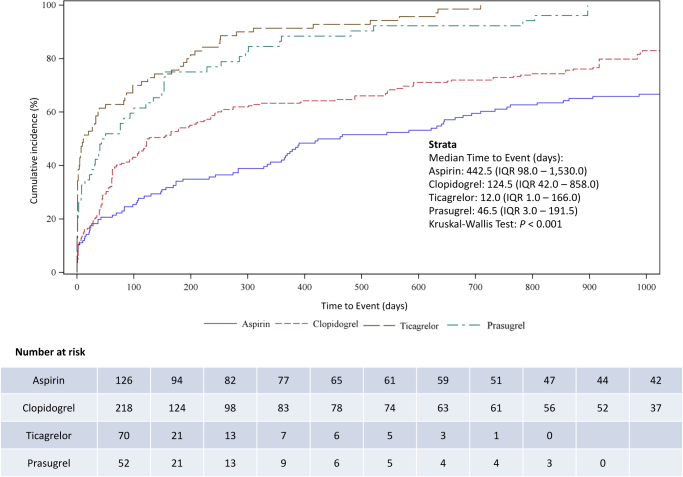
Comparison of cumulative incidence of CNS-related hematomas in patients receiving antiplatelet treatment in the FAERS database.

CNS, central nervous system; FAERS, the US Food and Drug Administration Adverse Event Reporting System; IQR, interquartile range.

TTO analysis was conducted to characterize the temporal distribution of CNS-related hematoma events associated with antiplatelet drugs. The median TTO varied significantly among the four antiplatelet agents (*P* < 0.001). Aspirin exhibited the longest latency period (median, 442.5 days; IQR, 98.0–1530.0 days), followed by clopidogrel (median, 124.5 days; IQR, 42.0–858.0 days), prasugrel (median, 46.5 days; IQR, 3.0–191.5 days), and ticagrelor (median, 12.0 days; IQR, 1.0–166.0 days) (Fig. 5).

The Weibull shape parameter (β) was <1 for all four drugs, with 95% CI <1 (Table 2), indicating an early failure-type pattern. This suggested that the risk of CNS-related hematomas was highest during the initial treatment period and decreased over time.

**Table 2:** Weibull shape parameter analysis of CNS-related hAE reports **across different antiplatelet drugs in the FAERS database**

Drugs	Case (*n*)	TTO	Weibull distribution		Type
		(days)	Scale parameter	Shape parameter	
		Median (IQR)	α (95% CI)	β (95% CI)	
Aspirin	126	442.5 (98.0–1530.0)	930.29 (686.12–1261.35)	0.62 (0.54–0.71)	Early failure
Clopidogrel	218	124.5 (42.0–858.0)	396.90 (310.88–506.73)	0.58 (0.53–0.65)	Early failure
Ticagrelor	70	12.0 (1.0–166.0)	66.32 (39.40–111.61)	0.51 (0.42–0.62)	Early failure
Prasugrel	52	46.5 (3.0–191.5)	137.12 (85.37–220.22)	0.65 (0.51–0.82)	Early failure

CI, confidence interval; CNS, central nervous system; FAERS, the US Food and Drug Administration Adverse Event Reporting System; hAE, hematoma adverse event; IQR, interquartile range; TTO, time-to-onset.

### Causal relationship global assessment

A global assessment based on the Bradford Hill criteria supported a likely causal association between CNS-related hematomas and the four antiplatelet drugs. This conclusion was confirmed by the strength of disproportionality signals (ROR ranging from 8.07 to 40.13), cross-validation of findings across different databases (FAERS and VigiAccess), multiple detection algorithms (ROR, PRR, BCPNN, and MGPS), and temporal patterns. Additionally, the biological plausibility of this association was supported by established pharmacological mechanisms of antiplatelet drugs (Table 3).

**Table 3:** Global assessment of a likely causal association between antiplatelet drugs and CNS-related hematomas using adapted Bradford Hill criteria

Criteria	Description	Source/method
Strength of the association	Strong disproportionate signals were observed for all four antiplatelet drugs across both databases, with ROR values ranging from 8.07 to 40.13. Consistent results were obtained across all detection algorithms. Warfarin (positive control) showed expectedly strong signals (FAERS: ROR, 38.31; VigiAccess: ROR, 41.08), while rosuvastatin (negative control) did not show significant signals (FAERS: ROR, 0.40; VigiAccess: ROR, 0.35)	Disproportionality analysis using ROR, PRR, BCPNN, and MGPS
Consistency	Results were consistent across databases (FAERS and VigiAccess) and detection algorithms. Positive and negative controls demonstrated expected patterns across databases	Cross-validation based on multiple detection algorithms and stratified analyses by age and gender. Comparative analysis with control drugs
Specificity	CNS-related hematoma subtypes showed specific strong associations with antiplatelet drugs, with subdural hematoma being the most common across all agents	PT-level analysis using BCPNN criteria, with IC025 > 0 as the signal detection threshold. Subdural hematoma was the most frequently reported subtype in both databases
Temporal relationship	An early failure-type pattern was identified for all four drugs, with the highest risk occurring during the initial treatment period. Median onset times varied significantly among different drugs	Time-to-onset analysis
Biological plausibility/empirical evidence	Antiplatelet drugs increase bleeding risk by inhibiting platelet aggregation^[24]^. Advanced age further increases susceptibility to hemorrhagic events^[25]^. Randomized controlled trials have documented increased bleeding risks with antiplatelet therapies, supporting our observations^[26]^	Descriptive analysis, established pharmacological mechanisms, and published clinical trials on antiplatelet therapy
Coherence	Findings align with established knowledge regarding the bleeding risks associated with antiplatelet therapy	Literature review and comparison with known safety profiles of antiplatelet drugs^[27]^
Analogy	Similar hemorrhagic risks have been observed with other antithrombotic agents (e.g., anticoagulants) in previous pharmacovigilance studies	Comparative evaluation with similar drug classes in pharmacovigilance literature^[28,29]^
Reversibility	Limited data are available on re-challenge and de-challenge events, reducing the relevance of this criterion in this study	Limitations of spontaneous reporting systems for capturing drug discontinuation outcomes

BCPNN, Bayesian confidence propagation neural network; CNS, central nervous system; FAERS, the US Food and Drug Administration Adverse Event Reporting System; IC, information component; MGPS, multi-item gamma Poisson shrinker; PRR, proportional reporting ratio; PT, preferred term; ROR, reporting odds ratio..

## Discussion

### Summary of key findings

This pharmacovigilance study analyzed data from the FAERS and VigiAccess databases, providing a comprehensive assessment of the association between antiplatelet drugs and CNS-related hematomas. Disproportionality analysis revealed significant safety signals for all four antiplatelet agents, with a clear scale of risk observed in the FAERS database: clopidogrel (ROR, 26.79) > aspirin (ROR, 22.06) > prasugrel (ROR, 16.91) > ticagrelor (ROR, 8.07). These results were replicated in the VigiAccess database, although signal strengths were generally higher: aspirin (ROR, 40.13) > clopidogrel (ROR, 36.69) > prasugrel (ROR, 24.02) > ticagrelor (ROR, 9.80). The robustness of our methodology was validated through the expected strong signal for warfarin (positive control) and the absence of significant signal for rosuvastatin (negative control) in both databases. We also identified demographic risk patterns and temporal characteristics that may influence clinical decision-making regarding antiplatelet therapy.

### Clinical significance of demographic patterns

In our analysis, males accounted for the majority of reported cases (57.81%) across all antiplatelet agents, which may reflect the higher prevalence of atherosclerotic cardiovascular disease in men^[30]^, leading to more frequent prescriptions of antiplatelet therapy^[31]^. However, stratified analysis revealed stronger signals in females compared to males across all four drugs. This apparent paradox – fewer female cases but stronger signal intensity – suggests that women may have a higher susceptibility to hemorrhage due to antiplatelet therapy. This finding is consistent with recent registry data, which showed a 2.33-fold greater adjusted bleeding risk in women receiving dual antiplatelet therapy, particularly at vascular access sites^[32]^. It highlights unique pathophysiological factors in women, which may affect platelet reactivity, vascular biology, and pharmacokinetics, thereby increasing their vulnerability to bleeding^[33]^. The stronger signals observed in female patients emphasize the importance of gender-specific considerations.

The age-stratified analysis revealed a complex relationship between age and the risk of CNS-related hematomas. While all age groups showed significant signals, the magnitude of the risk varied substantially. Elderly patients (≥65 years) accounted for the largest proportion of cases (68.48%), consistent with evidence that advanced age increases the risk of antithrombotic-related bleeding^[34]^. However, the age-stratified disproportionality analysis uncovered a more nuanced phenomenon: the 18–44 years cohort exhibited particularly strong signals for prasugrel (ROR, 133.20), suggesting that its hemorrhagic risk may be substantial even in populations typically considered at lower bleeding risk^[35,36]^.

By contrast, the elderly population (≥65 years) showed attenuated but still significant signals. These findings challenge the traditional view of a monotonically increasing bleeding risk with age, suggesting that age alone may inadequately predict antiplatelet-related hemorrhage. Clinicians should adopt more nuanced bleeding risk assessment tools, such as the PRECISE-DAPT or CRUSADE bleeding score, to guide antiplatelet therapy decisions^[37]^. Recent meta-analyses support the importance of individualized risk assessment beyond age; for instance, ticagrelor monotherapy has been shown to reduce major bleeding risk by 53% compared to standard DAPT without compromising ischemic outcomes^[38]^.

### Risk comparison with clinical trials

In the FAERS database, clopidogrel demonstrated the strongest association with CNS-related hematomas, followed by aspirin, prasugrel, and ticagrelor. These findings were replicated to some extent in the VigiAccess database. Notably, the risk hierarchy identified in our disproportionality analysis diverged from the bleeding risk profiles established in RCTs.

In the CAPRIE trial, clopidogrel demonstrated a comparable major bleeding rate to aspirin (intracranial 0.33% vs. 0.47%)^[39]^. In the TRITON-TIMI 38 trial, prasugrel exhibited a higher rate of major bleeding than clopidogrel (2.4% vs. 1.8%, hazard ratio: 1.32, *P* = 0.03), including life-threatening bleeding (1.4% vs. 0.9%, *P* = 0.01) and fatal bleeding (0.4% vs. 0.1%, *P* = 0.002)^[40]^. Similarly, in the PLATO trial, overall major bleeding rates were similar between ticagrelor and clopidogrel (11.6% vs. 11.2%, *P* = 0.43); however, ticagrelor was associated with a higher rate of major bleeding not related to coronary artery bypass grafting (4.5% vs. 3.8%, *P* = 0.03)^[6]^. In the ISAR-REACT 5 trial, the major bleeding rates were comparable between prasugrel and ticagrelor (4.8% vs. 5.4%; hazard ratio: 1.12, *P* = 0.46)^[5]^.

Several factors may explain these differences. First, RCTs follow standardized protocols for dosing and concomitant medications, whereas real-world data involve variable dosing regimens, off-label use, and combination therapies, all of which can affect bleeding risk^[41]^. Second, aspirin and clopidogrel have been on the market longer than ticagrelor and prasugrel, leading to a larger cumulative number of reported cases in real-world settings. Third, RCTs typically focus on composite bleeding outcomes, such as International Society on Thrombosis and Hemostasis or Thrombolysis in Myocardial Infarction bleeding criteria^[42]^, whereas our analysis focused on CNS-related hematomas, emphasizing risk assessment based on bleeding subtypes, particularly in high-risk populations. Last, the risk hierarchy for CNS-related hemorrhage may differ from overall bleeding events due to drug-specific factors, such as blood–brain barrier penetration or cerebrovascular distribution^[43]^.

Recent meta-analyses provide additional supplements for interpreting our findings. A meta-analysis involving 14 545 patients found that compared with patients not on antithrombotic therapy, those on antiplatelet therapy had a higher risk of ICH after a mild traumatic brain injury [RR (risk ratio), 1.51]^[44]^. Another meta-analysis showed that antiplatelet therapy in patients with head injury was associated with an increased risk of traumatic ICH (odds ratio, 1.87), with the risk being higher in patients with mild traumatic brain injury (odds ratio, 2.72)^[45]^. Our findings extend these observations to newer antiplatelet agents and provide in-depth insights into specific hematoma subtypes.

### PT-level analysis

At the PT level, subdural hematoma was the most frequently reported CNS-related hAE across all four antiplatelet drugs (FAERS, 63.11%; VigiAccess, 62.72%). This predominance is pathophysiologically plausible. Subdural hematomas primarily result from the rupture of bridging veins that traverse the subdural space^[46]^, and these veins are particularly vulnerable to shearing forces, even during minor trauma. Pharmacologically, by blocking various platelet activation pathways, antiplatelet therapy may impair effective hemostasis following vessel rupture^[47]^. In our study, elderly patients accounted for the majority, whose age-related cerebral atrophy may increase the likelihood of bridging venous injury^[48]^.

Spinal epidural hematoma demonstrated particularly strong signals for aspirin (FAERS: IC025, 4.11; VigiAccess: IC025, 4.97) and clopidogrel (FAERS: IC025, 5.25; VigiAccess: IC025, 5.54). Although less common than intracranial hematomas, spinal epidural hematomas are potentially catastrophic, as they can lead to permanent paraplegia due to spinal cord ischemia and infarction if not promptly diagnosed and treated^[49]^. Spinal epidural hematoma risk is amplified in patients with modifiable risk factors, such as obesity or hypertension, as demonstrated by a recent meta-analysis^[50]^. This strong signal, which has received limited attention in RCTs of antiplatelet agents, represents a critical finding of our pharmacovigilance analysis.

Cerebral hematoma also showed robust signals across all four antiplatelet agents, particularly for aspirin (FAERS: IC025 3.27; VigiAccess: IC025 5.27) and clopidogrel (FAERS: IC025 4.00; VigiAccess: IC025 4.56). This finding is particularly significant, given that intraparenchymal hemorrhage generally carries a poorer prognosis than other CNS-related hematoma subtypes, with high mortality and disability rates^[51]^. The signal for this severe complication underscores the importance of careful risk-benefit assessment before initiating antiplatelet therapy.

The differences in hematoma subtypes across the drugs may reflect variations in their pharmacological properties and the patient populations. This distinction is mechanistically illustrated by thienopyridines (clopidogrel and prasugrel), which achieve irreversible P2Y12 receptor blockade through active metabolites, contrasting with the direct-acting and reversible inhibition for ticagrelor^[3]^. Additionally, aspirin and clopidogrel are more frequently used in patients with peripheral arterial disease^[52]^, who are more likely to have concomitant spinal pathology, potentially explaining the stronger signal for spinal hematomas.

### Temporal pattern analysis

All four drugs exhibited an early failure pattern, underscoring the need for more intensive monitoring during the initial weeks to months of therapy, particularly for ticagrelor (median, 12.0 days) and prasugrel (median, 46.5 days), which demonstrated the shortest latency periods. These findings are consistent with clinical trial data. In the THALES trial, an increase in major hemorrhage with ticagrelor was observed during the first week, with the risk remaining relatively constant in the subsequent weeks^[53]^. Similarly, a network meta-analysis of RCTs showed that early prasugrel significantly reduced major adverse cardiovascular events compared with early clopidogrel, but at the cost of increased bleeding risk^[54]^.

Our findings provide real-world validation of these clinical trials and extend them to more specific CNS-related hematoma events. During this high-risk period, health care providers should consider neuroimaging for patients presenting with neurological symptoms, such as severe headaches, altered mental status, or focal neurological deficits^[55]^.

### Biological plausibility and mechanistic explanations

Aspirin irreversibly inhibits cyclooxygenase-1, blocking thromboxane A2 and subsequent platelet aggregation^[56]^. Thienopyridines (clopidogrel and prasugrel) irreversibly bind to the P2Y12 receptor, whereas ticagrelor binds reversibly to this receptor^[56]^. These mechanistic differences are likely contributors to the divergent risk signals observed in our analysis.

Theoretically, the reversibility of platelet inhibition by ticagrelor may provide an advantage in hemorrhagic scenarios. With a half-life of approximately 8–12 hours, platelet function can partially recover between doses, potentially allowing for more effective hemostasis in the event of vascular injury. In contrast, the irreversible inhibition induced by clopidogrel and prasugrel requires the production of new platelets to restore hemostatic function, a process that takes approximately 7–10 days^[57]^. This pharmacological distinction may partially explain the lower signal strength observed with ticagrelor, despite its potent antiplatelet effect.

### Strengths and limitations

Our study has several strengths, including the large sample size from two global pharmacovigilance databases, the use of multiple disproportionality algorithms for signal detection, and the inclusion of appropriate positive and negative controls. The PT-level analysis, TTO data, and causal relationship assessment provide a strong evidence hierarchy, which deserves further validation in future antiplatelet therapy research.

However, several limitations must be acknowledged. Spontaneous reporting systems are subject to reporting biases, including underreporting, selective reporting, media attention, regulatory warnings, and cognitive biases. Moreover, the databases do not always include critical clinical information, such as the severity of the disease, antiplatelet drug dosage, treatment duration, comorbidities, patient-specific risk factors for hemorrhage, or drug compliance data, limiting the depth of analysis regarding these risk factors. Additionally, the absence of reliable denominator data makes it impossible to calculate true incidence rates. Finally, disproportionality analyses cannot quantify risk or prove causality, but only count the strength of the association. Future research should address these limitations to bridge the gap between drug warning signals and clinical trial data.

## Conclusions

This pharmacovigilance analysis indicates significant associations between antiplatelet drugs and CNS-related hematomas, with distinct patterns observed across drugs, patient demographics, hematoma subtypes, and time course. Our findings provide valuable insights into the safety profile of antiplatelet agents, particularly during high-risk periods and in vulnerable populations. Future research should prioritize the development of personalized treatment strategies to improve clinical outcomes for patients receiving antiplatelet therapy.

## Supplementary Material

## Data Availability

The datasets generated and/or analyzed during the current study can be available in the US FAERS database (https://fis.fda.gov/extensions/fpd-qde-faers/fpd-qde-faers.html) and the VigiAccess database (https://www.vigiaccess.org). The dataset used in the study can be made available from the corresponding author upon request.
