# Supplementary material for: Antiplatelet therapy and central nervous system hematomas: a cohort study using real-world data from the FAERS and VigiAccess databases
Source: Int J Surg. 2025 Sep 8;112(1):1120–32. doi: 10.1097/JS9.0000000000003421 (PMC12825548; doi:10.1097/JS9.0000000000003421)
Supplement: Supplementary file 1 [file js9-112-1120-001.docx]

# SUPPORTING INFORMATION

# Additional Tables

**Table S1 Details of PTs, HLTs, and HLGTs were included in SOCs of CNS-related hAE reports in MedDRA (Version 27.1)**

**Table S2 Four table of measure of disproportionality**

**Table S3 ROR, PRR, BCPNN and MGPS methods, formulas, and thresholds**

**Table S4 Signals identification of CNS-related hAEs across the four antiplatelet drugs using disproportionality analysis in the two databases**

**Table S5 Number of cases of PTs for CNS-related hAE reports across the four antiplatelet drugs in the two databases**

**Table S6 PTs for CNS-related hAE reports using BCPNN criteria across all four antiplatelet drugs in the two databases**

**Table S7 Stratified analysis of CNS-related hAE reports by age and gender across different antiplatelet drugs in the FAERS database**

## Table S1 Details of PTs, HLTs, and HLGTs were included in SOCs of CNS-related hAE reports in MedDRA (Version 27.1)

| **PT code** | **PT name** | **HLT code** | **HLT name** | **HLGT code** | **HLGT name** | **SOC code** | **SOC name** |
| --- | --- | --- | --- | --- | --- | --- | --- |
| 10061038 | Cerebellar haematoma | 10007948 | Central nervous system haemorrhages and cerebrovascular accidents | 10007963 | Central nervous system vascular disorders | 10029205 | Nervous system disorders |
| 10015769 | Extradural haematoma | 10044518 | Traumatic central nervous system haemorrhages | 10007963 | Central nervous system vascular disorders | 10029205 | Nervous system disorders |
| 10053942 | Cerebral haematoma | 10007948 | Central nervous system haemorrhages and cerebrovascular accidents | 10007963 | Central nervous system vascular disorders | 10029205 | Nervous system disorders |
| 10042361 | Subdural haematoma | 10044518 | Traumatic central nervous system haemorrhages | 10007963 | Central nervous system vascular disorders | 10029205 | Nervous system disorders |
| 10050162 | Spinal epidural haematoma | 10007948 | Central nervous system haemorrhages and cerebrovascular accidents | 10007963 | Central nervous system vascular disorders | 10029205 | Nervous system disorders |
| 10050164 | Spinal subdural haematoma | 10007948 | Central nervous system haemorrhages and cerebrovascular accidents | 10007963 | Central nervous system vascular disorders | 10029205 | Nervous system disorders |
| 10059491 | Intracranial haematoma | 10007948 | Central nervous system haemorrhages and cerebrovascular accidents | 10007963 | Central nervous system vascular disorders | 10029205 | Nervous system disorders |
| 10073230 | Brain stem haematoma | 10007948 | Central nervous system haemorrhages and cerebrovascular accidents | 10007963 | Central nervous system vascular disorders | 10029205 | Nervous system disorders |
| 10076051 | Spinal cord haematoma | 10007948 | Central nervous system haemorrhages and cerebrovascular accidents | 10007963 | Central nervous system vascular disorders | 10029205 | Nervous system disorders |
| 10076701 | Subarachnoid haematoma | 10007948 | Central nervous system haemorrhages and cerebrovascular accidents | 10007963 | Central nervous system vascular disorders | 10029205 | Nervous system disorders |
| 10077031 | Basal ganglia haematoma | 10007948 | Central nervous system haemorrhages and cerebrovascular accidents | 10007963 | Central nervous system vascular disorders | 10029205 | Nervous system disorders |
| 10079013 | Traumatic intracranial haematoma | 10044518 | Traumatic central nervous system haemorrhages | 10007963 | Central nervous system vascular disorders | 10029205 | Nervous system disorders |
| 10080347 | Extraischaemic cerebral haematoma | 10007948 | Central nervous system haemorrhages and cerebrovascular accidents | 10007963 | Central nervous system vascular disorders | 10029205 | Nervous system disorders |
| 10090938 | Spontaneous subdural haematoma | 10007948 | Central nervous system haemorrhages and cerebrovascular accidents | 10007963 | Central nervous system vascular disorders | 10029205 | Nervous system disorders |
| 10008014 | Cephalhaematoma | 10018987 | Haemorrhages NEC | 10047075 | Vascular haemorrhagic disorders | 10047065 | Vascular disorders |

Abbreviations: PT, preferred term; PT code, distinct identify of PT in the Medical Dictionary for Regulatory Activities (MedDRA); HLT, high-level term; HLT code, distinct identify of HLT in the MedDRA; HLGT, high-level group term; HLGT code, distinct identify of HLGT in the MedDRA; SOC, system organ class; SOC code, distinct identify of SOC in the MedDRA; CNS, central nervous system; hAE, hematoma adverse event.

## Table S2 Four table of measure of disproportionality

|  | **Target AEs reported** | **Non-target AEs reported** | **Total** |
| --- | --- | --- | --- |
| Antiplatelet drugs | a | b | a+b |
| Non- Antiplatelet drugs | c | d | c+d |
| Total | a+c | b+d | a+b+c+d |

a = the number of reports of antiplatelet drugs with the adverse event of interest.

b = the number of reports of all other drugs with the adverse event of interest.

c = the number of reports of antiplatelet drugs with all other adverse events.

d = the number of reports of all other drugs with all other adverse events.

Abbreviations: AE, adverse event.

## Table S3 ROR, PRR, BCPNN and MGPS methods, formulas, and thresholds

| **Method** | **Calculation formula** | **Threshold** |
| --- | --- | --- |
| ROR | 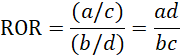    95%CI=*e*^1n(ROR)±1.96SE^ | a ≥ 3  95% CI (lower limit) > 1 |
| PRR | $PRR=\frac{\text{a}/\text{（}a+b\text{）}}{c/(c+d)}$ $SE\text{（}1\text{n}PRR\text{）}=\sqrt{(\frac{1}{\text{a}}-\frac{1}{a+b}+\frac{1}{c}-\frac{1}{c+d})}$    95%CI=*e*^1n(PRR)±1.96SE^ | a ≥ 3  95% CI (lower limit) > 1 |
| BCPNN | $IC=$ 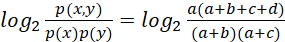  E(IC)=${log}_{2}\frac{(a+\gamma11)(a+b+c+d+\alpha)(a+b+c+d+\beta)}{（a+b+c+d+\gamma）(a+b+\alpha1)(a+c+\beta1)}$  V(IC) = 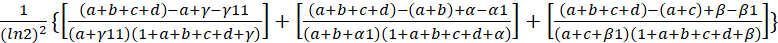  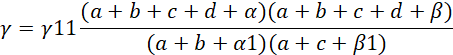    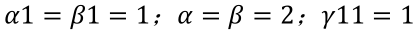 | IC025 > 0  Low Signal (+): 0 < IC025 ≤ 1.5  Medium Signal (++): 1.5 < IC025 ≤ 3  High Signal (+++): IC025 > 3 |
| MGPS | 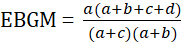  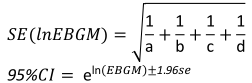 | EBGM05 > 2  a > 0 |

a = the number of reports of antiplatelet drugs with the adverse event of interest.

b = the number of reports of all other drugs with the adverse event of interest.

c = the number of reports of antiplatelet drugs with all other adverse events.

d = the number of reports of all other drugs with all other adverse events.

Abbreviations: ROR, reporting odds ratio; PRR, proportional reporting ratio; BCPNN, bayesian confidence propagation neural network; MGPS, multi-item gamma Poisson shrinker; EBGM, empirical Bayesian geometric mean; CI, confidence interval; IC, information component; IC025, the lower limit of the 95% one-sided CI, of the IC; EBGM05, the lower 95% one-sided CI, of EBGM.

## Table S4 Signals identification of CNS-related hAEs across the four antiplatelet drugs using disproportionality analysis in the two databases

| Drugs | AE Reports | ROR  (95% Cl） | PRR  (95% Cl） | BCPNN  IC (IC025) | MGPS  EBGM (EBGM05) |
| --- | --- | --- | --- | --- | --- |
| **FAERS** |  |  |  |  |  |
| Aspirin | 952 | 22.06 (20.66, 23.55) | 21.90 (20.53, 23.37) | 4.38 (4.26) | 20.88 (19.56) |
| Clopidogrel | 1,076 | 26.79 (25.19, 28.50) | 26.56 (24.98, 28.24) | 4.65 (4.53) | 25.15 (23.65) |
| Ticagrelor | 145 | 8.07 (6.86, 9.51) | 8.05 (6.84, 9.48) | 3.00 (2.69) | 8.00 (6.79) |
| Prasugrel | 101 | 16.91 (13.90, 20.57) | 16.81 (13.83, 20.43) | 4.06 (3.57) | 16.73 (13.75) |
| Warfarin  (Positive control) | 1516 | 38.31 (36.35, 40.39) | 37.85 (35.93, 39.88) | 5.13 (5.02) | 34.99 (33.20) |
| Rosuvastatin  (Negative control) | 20 | 0.40 (0.26, 0.62) | 0.40 (0.26, 0.62) | -1.32 (-1.91) | 0.40 (0.26) |
| **VigiAccess** |  |  |  |  |  |
| Aspirin | 4,867 | 40.13 (38.94, 41.37) | 39.71 (38.53, 40.92) | 5.11 (5.06) | 34.63 (33.60) |
| Clopidogrel | 1,998 | 36.69 (35.06, 38.39) | 36.30 (34.71, 37.97) | 5.10 (5.01) | 34.40 (32.88) |
| Ticagrelor | 203 | 9.80 (8.54, 11.25) | 9.77 (8.52, 11.22) | 3.28 (3.02) | 9.73 (8.47) |
| Prasugrel | 161 | 24.02 (20.57, 28.06) | 23.85 (20.44, 27.83) | 4.57 (4.15) | 23.75 (20.33) |
| Warfarin  (Positive control) | 3,321 | 41.08 (39.64, 42.58) | 40.61 (39.20, 42.08) | 5.21 (5.14) | 37.07 (35.77) |
| Rosuvastatin  (Negative control) | 22 | 0.35 (0.23, 0.54) | 0.35 (0.23, 0.54) | -1.50 (-2.06) | 0.35 (0.23) |

Abbreviations: CNS, central nervous system; hAE, hematoma adverse event; AE, adverse event; FAERS, the U.S. Food and Drug Administration Adverse Event Reporting System; ROR, reporting odds ratio; CI, confidence interval; PRR, proportional reporting ratio; BCPNN, Bayesian confidence propagation neural network; IC, information component; MGPS, multi-item gamma Poisson shrinker; EBGM, empirical Bayesian geometric mean.

## Table S5 Number of cases of PTs for CNS-related hAE reports across the four antiplatelet drugs in the two databases

| PTs (n) | Total reports | Aspirin | Clopidogrel | Ticagrelor | Prasugrel |
| --- | --- | --- | --- | --- | --- |
| **FAERS** | 2,274 |  |  |  |  |
| Subdural hematoma | 1,435 | 630 | 639 | 88 | 78 |
| Cerebral hematoma | 284 | 96 | 146 | 31 | 11 |
| Extradural hematoma | 206 | 99 | 97 | 6 | 4 |
| Spinal epidural hematoma | 152 | 49 | 98 | 1 | 4 |
| Intracranial hematoma | 56 | 27 | 22 | 5 | 2 |
| Spinal cord hematoma | 40 | 15 | 19 | 6 | 0 |
| Spinal subdural hematoma | 27 | 11 | 16 | 0 | 0 |
| Cerebellar hematoma | 30 | 11 | 13 | 4 | 2 |
| Subarachnoid hematoma | 33 | 8 | 23 | 2 | 0 |
| Brain stem hematoma | 4 | 3 | 0 | 1 | 0 |
| Traumatic intracranial hematoma | 2 | 2 | 0 | 0 | 0 |
| Basal ganglia hematoma | 3 | 1 | 2 | 0 | 0 |
| Cephalhematoma | 2 | 0 | 1 | 1 | 0 |
| **VigiAccess database** | 7,229 |  |  |  |  |
| Subdural hematoma | 4,534 | 3,013 | 1,282 | 122 | 117 |
| Cerebral hematoma | 1,482 | 1,114 | 310 | 41 | 17 |
| Extradural hematoma | 415 | 244 | 151 | 9 | 11 |
| Spinal epidural hematoma | 276 | 145 | 123 | 5 | 3 |
| Intracranial hematoma | 203 | 137 | 50 | 11 | 5 |
| Spinal cord hematoma | 74 | 42 | 24 | 7 | 1 |
| Spinal subdural hematoma | 34 | 25 | 6 | 2 | 1 |
| Cerebellar hematoma | 129 | 98 | 25 | 4 | 2 |
| Subarachnoid hematoma | 40 | 19 | 18 | 1 | 2 |
| Brain stem hematoma | 18 | 13 | 3 | 1 | 1 |
| Traumatic intracranial hematoma | 7 | 6 | 1 | 0 | 0 |
| Basal ganglia hematoma | 9 | 6 | 2 | 0 | 1 |
| Cephalhematoma | 8 | 5 | 3 | 0 | 0 |

Abbreviations: PT, preferred term; CNS, central nervous system; hAE, hematoma adverse event; FAERS, the U.S. Food and Drug Administration Adverse Event Reporting System.

## Table S6 PTs for CNS-related hAE reports using BCPNN criteria across all four antiplatelet drugs in the two databases

| PTs | Aspirin  IC (IC025) | Clopidogrel  IC (IC025) | Ticagrelor  IC (IC025) | Prasugrel  IC (IC025) |
| --- | --- | --- | --- | --- |
| **FAERS database** |  |  |  |  |
| Subdural hematoma | 4.36 (4.19) | 4.47 (4.30) | 2.85 (2.44) | 4.26 (3.63) |
| Cerebral hematoma | 3.74 (3.27) | 4.44 (4.00) | 3.44 (2.54) | 3.53 (1.79) |
| Extradural hematoma | 5.09 (4.38) | 5.15 (4.41) | 2.38 (0.60) | 3.38 (0.56) |
| Spinal epidural hematoma | 5.41 (4.11) | 6.51 (5.25) | 1.13 (-1.59)^#^ | 4.72 (0.82) |
| Intracranial hematoma | 4.46 (3.09) | 4.25 (2.80) | 3.36 (0.83) | 3.62 (-0.30)^#^ |
| Spinal cord hematoma | 4.42 (2.49) | 4.85 (2.92) | 4.43 (1.35) | — |
| Subarachnoid hematoma | 4.79 (1.81) | 6.41 (3.59) | 4.12 (-0.26)^#^ | — |
| Cerebellar hematoma | 4.09 (2.01) | 4.42 (2.33) | 3.96 (0.69) | 4.55 (-0.21)^#^ |
| Spinal subdural hematoma | 5.33 (2.36) | 5.97 (3.01) | — | — |
| Brain stem hematoma | 4.87 (0.35) | — | 4.62 (-1.14)^#^ | — |
| Traumatic intracranial hematoma | 5.16 (-0.26)^#^ | — | — | — |
| Basal ganglia hematoma | 3.70 (-1.21)^#^ | 4.79 (-0.26)^#^ | — | — |
| Cephalhematoma | — | 2.19 (-1.35)^#^ | 3.43 (-1.19)^#^ | — |
| **VigiAccess database** |  |  |  |  |
| Subdural hematoma | 5.01 (4.94) | 5.06 (4.94) | 3.14 (2.79) | 4.70 (4.16) |
| Cerebral hematoma | 5.42 (5.27) | 4.85 (4.56) | 3.41 (2.66) | 3.76 (2.31) |
| Extradural hematoma | 5.01 (4.64) | 5.59 (4.96) | 3.00 (1.32) | 4.91 (2.30) |
| Spinal epidural hematoma | 5.66 (4.97) | 6.70 (5.54) | 3.55 (0.89) | 4.44 (0.37) |
| Intracranial hematoma | 5.26 (4.65) | 5.08 (3.94) | 4.37 (2.13) | 4.85 (1.17) |
| Cerebellar hematoma | 5.27 (4.50) | 4.58 (3.09) | 3.41 (0.57) | 4.03 (-0.25)^#^ |
| Spinal cord hematoma | 4.66 (3.55) | 5.13 (3.30) | 4.83 (1.65) | 3.64 (-1.16)^#^ |
| Subarachnoid hematoma | 4.64 (2.82) | 5.83 (3.16) | 3.14 (-1.21)^#^ | 5.76 (-0.15)^#^ |
| Spinal subdural hematoma | 5.29 (3.38) | 4.50 (1.35) | 4.40 (-0.23)^#^ | 5.02 (-1.10)^#^ |
| Brain stem hematoma | 5.42 (2.59) | 4.58 (0.35) | 4.48 (-1.13)^#^ | 6.10 (-1.09)^#^ |
| Basal ganglia hematoma | 4.46 (1.31) | 4.15 (-0.27)^#^ | — | 6.25 (-1.09)^#^ |
| Cephalhematoma | 2.58 (0.51) | 3.12 (0.11) | — | — |
| Traumatic intracranial hematoma | 5.42 (1.43) | 4.11 (-1.18)^#^ | — | — |

Note: #: the values that did not meet the threshold for a positive signal of BCPNN criteria.

Abbreviations: PT, preferred term; CNS, central nervous system; hAE, hematoma adverse event; BCPNN, Bayesian confidence propagation neural network; IC, information component; FAERS, the U.S. Food and Drug Administration Adverse Event Reporting System.

## Table S7 Stratified analysis of CNS-related hAE reports by age and gender across different antiplatelet drugs in the FAERS database

| Stratification | Drug | Reports | ROR  (95% CI) | PRR  (95% CI) | BCPNN  (IC025) | EBGM  (EBGM05) |
| --- | --- | --- | --- | --- | --- | --- |
| Male | Aspirin | 568 | 20.41 (18.75, 22.23) | 20.21 (18.58, 21.99) | 4.09 | 19.14 (17.58) |
|  | Clopidogrel | 589 | 19.72 (18.14, 21.44) | 19.54 (17.98, 21.22) | 4.04 | 18.46 (16.98) |
|  | Ticagrelor | 101 | 6.53 (5.37, 7.95) | 6.52 (5.36, 7.92) | 2.33 | 6.46 (5.31) |
|  | Prasugrel | 67 | 11.83 (9.30, 15.05) | 11.76 (9.26, 14.94) | 2.98 | 11.69 (9.19) |
| Female | Aspirin | 317 | 26.16 (23.37, 29.29) | 26.01 (23.25, 29.09) | 4.37 | 24.98 (22.31) |
|  | Clopidogrel | 334 | 31.68 (28.38, 35.37) | 31.46 (28.20, 35.09) | 4.63 | 30.13 (26.99) |
|  | Ticagrelor | 41 | 8.67 (6.38, 11.79) | 8.65 (6.37, 11.76) | 2.42 | 8.61 (6.33) |
|  | Prasugrel | 31 | 21.35 (14.99, 30.41) | 21.25 (14.94, 30.21) | 3.19 | 21.16 (14.86) |
| < 18 y | Aspirin | 12 | 22.57 (12.68, 40.19) | 22.49 (12.66, 39.97) | 2.25 | 21.75 (12.22) |
|  | Clopidogrel | 0 | — | — | — | — |
|  | Ticagrelor | 0 | — | — | — | — |
|  | Prasugrel | 0 | — | — | — | — |
| 18–44 y | Aspirin | 36 | 23.83 (17.08, 33.24) | 23.76 (17.05, 33.11) | 3.37 | 22.99 (16.48) |
|  | Clopidogrel | 10 | 27.20 (14.58, 50.76) | 27.11 (14.56, 50.48) | 2.13 | 26.86 (14.40) |
|  | Ticagrelor | 2 | 14.63 (3.65, 58.66) | 14.61 (3.65, 58.40) | -0.27 | 14.58 (3.64) |
|  | Prasugrel | 8 | 133.20 (66.05, 268.65) | 130.96 (65.71, 261.03) | 2.11 | 129.99 (64.45) |
| 45–64 y | Aspirin | 147 | 26.17 (22.17, 30.89) | 26.02 (22.06, 30.68) | 4.18 | 24.86 (21.06) |
|  | Clopidogrel | 121 | 22.76 (18.97, 27.30) | 22.64 (18.89, 27.14) | 3.95 | 21.82 (18.19) |
|  | Ticagrelor | 41 | 13.42 (9.86, 18.28) | 13.38 (9.84, 18.21) | 2.91 | 13.23 (9.71) |
|  | Prasugrel | 35 | 29.70 (21.26, 41.48) | 29.49 (21.16, 41.10) | 3.55 | 29.18 (20.89) |
| ≥ 65 y | Aspirin | 666 | 13.48 (12.46, 14.58) | 13.33 (12.33, 14.41) | 3.52 | 12.61 (11.66) |
|  | Clopidogrel | 763 | 13.86 (12.88, 14.93) | 13.71 (12.74, 14.75) | 3.55 | 12.86 (11.95) |
|  | Ticagrelor | 86 | 4.57 (3.70, 5.66) | 4.56 (3.69, 5.63) | 1.81 | 4.53 (3.66) |
|  | Prasugrel | 44 | 8.15 (6.06, 10.98) | 8.10 (6.03, 10.88) | 2.37 | 8.07 (6.00) |

Abbreviation: CNS, central nervous system; hAE, hematoma adverse event; FAERS, the U.S. Food and Drug Administration Adverse Event Reporting System; ROR, reporting odds ratio; CI, confidence interval; PRR, proportional reporting ratio; BCPNN, Bayesian confidence propagation neural network; IC, information component; MGPS, multi-item gamma Poisson shrinker; EBGM, empirical Bayesian geometric mean.
